# Comprehensive genomic and immunological characterization of Chinese non-small cell lung cancer patients

**DOI:** 10.1038/s41467-019-09762-1

**Published:** 2019-04-16

**Authors:** Xu-Chao Zhang, Jun Wang, Guo-Guang Shao, Qun Wang, Xiaotao Qu, Bo Wang, Christopher Moy, Yue Fan, Zayed Albertyn, Xiayu Huang, Jingyu Zhang, Yang Qiu, Suso Platero, Matthew V. Lorenzi, Enrique Zudaire, Jennifer Yang, Ying Cheng, Lin Xu, Yi-Long Wu

**Affiliations:** 1 Guangdong Lung Cancer Institute, Guangdong Provincial People’s Hospital and Guangdong Academy of Medical Sciences, 510080 Guangzhou, China; 2 Peking University People’s Hospital, Beijing, 100044 China; 3 Thoracic Surgery, 1st Hospital of Jilin University, 130021 Changchun, China; 4 Zhongshan Hospital, Fudan University, 200032 Shanghai, China; 5 Janssen R&D China, 355 Hong Qiao Road, 200030 Shanghai, China; 6 Janssen R&D, 1400 McKean Road, Spring House, Pennsylvania 19002 USA; 7 Novocraft Technologies, 46300 Petaling Jaya, Selangor Malaysia; 8 Department of Medical Oncology, Jilin Provincial Cancer Hospital, 130012 Changchun, China; 9 Department of Thoracic Surgery, Nanjing Medical University Affiliated Cancer Hospital, Jiangsu Key Laboratory of Molecular and Translational Cancer Research, Cancer Institute of Jiangsu Province, 210009 Nanjing, China

## Abstract

Deep understanding of the genomic and immunological differences between Chinese and Western lung cancer patients is of great importance for target therapy selection and development for Chinese patients. Here we report an extensive molecular and immune profiling study of 245 Chinese patients with non-small cell lung cancer. Tumor-infiltrating lymphocyte estimated using immune cell signatures is found to be significantly higher in adenocarcinoma (ADC, 72.5%) compared with squamous cell carcinoma (SQCC, 54.4%). The correlation of genomic alterations with immune signatures reveals that low immune infiltration was associated with *EGFR* mutations in ADC samples, PI3K and/or WNT pathway activation in SQCC. While *KRAS* mutations are found to be significantly associated with T cell infiltration in ADC samples. The SQCC patients with high antigen presentation machinery and cytotoxic T cell signature scores are found to have a prolonged overall survival time.

## Abstract (web summary)

The relationship between genomic alteration and immune context in non-small cell lung cancer (NSCLC) is complex. Here, the authors analyse the molecular and immunological landscape of 245 Chinese patients with NSCLC and find low immune infiltration correlates with genomic alterations.

## Introduction

Lung cancer is among the most frequently diagnosed cancer and the leading cause of cancer-related mortality^1^. Non-small cell lung cancer (NSCLC) contributes 75% of lung cancer with most patients diagnosed in the advanced stage. The 5-year survival rate for NSCLC is only 15%. In 2015, a total of 733,300 new lung cancer cases and 610,200 lung cancer deaths were estimated in China^2^. It remains a significant unmet medical need and a huge burden on the healthcare system.

Targeted therapies have been successfully developed to treat NSCLC patients harboring driver gene mutations. Genomics studies have revealed distinct genetic mutation profiles for adenocarcinoma (ADC) and squamous cell carcinoma (SQCC)^3,4^. High prevalence of driver gene mutations and fusions in *EGFR*, *ALK*, *RET*, *ROS1*, and *KRAS* in lung ADC patients, while more frequent *PIK3CA*, *AKT1*, and *CDKN2A* mutations have been observed in lung SQCC patients^3,4^. Notably, non-smoker East Asian women are more likely to develop ADC and exhibit a higher incidence of *EGFR* mutation and a lower *KRAS* mutation frequency^5,6^. Further efforts are required to identify additional genomic alterations in Asian lung cancer patient population.

In recent years, immunotherapy, especially immune checkpoints blockage treatment such as PD-1 and PD-L1 inhibitors have been approved as first- or second-line treatment for various lung cancer types^7^. Targeting LAG3, TIM3, Tregs, and immunosuppressive factors released by Tregs (e.g., TGF-β) into the tumor microenvironment (TME) have also been proposed as additional strategies to re-establish the antitumoral immune response^8^.

To better prioritize treatment options and develop a more comprehensive picture of the TME, we carried out the CHOICE study to perform extensive molecular and immune annotation in 245 Chinese NSCLC patients. To identify additional genomic targets that are enriched in the Chinese population, we compared the genomic data with The Cancer Genome Atlas (TCGA) dataset. The relationship between the immune cell composition in TME and tumor genomic alteration in the Chinese NSCLC patients was investigated using the immune cell signatures. The prognostic role of the immune cell signatures was also evaluated.

## Results

### Samples and clinical data description

Tumor samples and adjacent normal tissues were obtained from 245 treatment-naïve NSCLC patients (ADC: 131, SQCC: 114). The peripheral blood was also collected to generate germline variants. The detailed patient clinical information can be found in the Supplementary Data 1. The mean age of the patients was 61.5 (SD = 9.00) years for ADC and 63.0 (SD = 7.15) years for SQCC (Table 1). Total 64% (*n* = 84/131) of ADC patients and 97% (*n* = 111/114) of SQCC patients had a history of tobacco use. The DNA and RNA were extracted and processed for genomics analysis as outlined in the online methods.

**Table 1:** Patient demographics

	ADC (*n* = 131)	SQCC (*n* = 114)
Age, mean (SD)	61.5 (9.00)	63.0 (7.15)
Gender^a^		
Men	91 (69)	109 (96)
Women	37 (28)	4 (4)
Unknown	3 (2)	1 (1)
Smoking status^a^		
No	47 (36)	3 (3)
Yes	84 (64)	111 (97)
Tumor stage^a^		
I	69 (53)	40 (35)
II	26 (20)	46 (40)
III	29 (22)	27 (24)
IV	7 (5)	1 (1)

^a^All values refer to percentage

### Somatic copy number variation

**Fig. 1:**
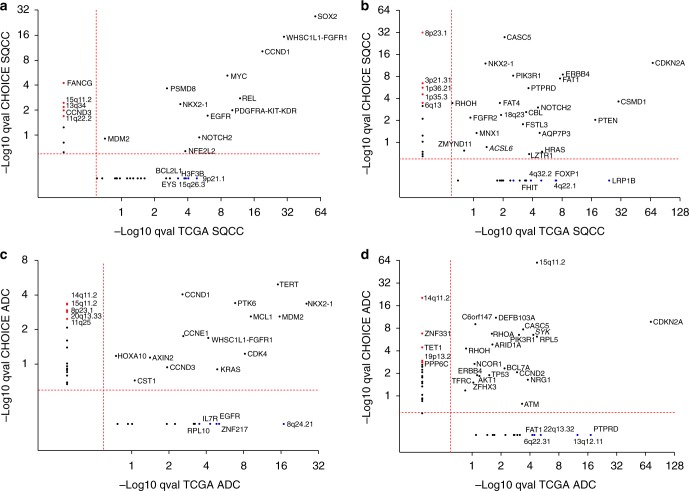
Copy number variations in the CHOICE versus TCGA NSCLC data. **a** SQCC focal amplifications. **b** SQCC focal deletions. **c** ADC focal amplifications. **d** ADC focal deletions. The GISTIC 2.0 algorithm was used to identify significantly recurrent copy number gains and losses in ADC and SQCC population, respectively. The *q* values for amplification (**a**, **c**) and deletions (**b**, **d**) in the CHOICE study were plotted against the *q* values from the TCGA datasets. CNVs with *q* values <0.25 were significant. Representative genes in focal regions were used whenever possible. Red dots: significant focal events only in the CHOICE cohort (ADC); blue dots: significant focal events only in TCGA dataset (SQCC). Red dashed line: *q* value cutoffs. Source data are provided as a Source Data file

The GISTIC 2.0 algorithm was used to identify significantly recurrent focal copy number gains and losses in ADC and SQCC patients, and the resulting *q* values were compared with the respective *q* values from the TCGA patients^3,4^. The overall copy number variation (CNV) profiles in the CHOICE study demonstrated distinct patterns for different lung cancer subtypes (Supplementary Fig. 1, Supplementary Data 2). Arm level gains of 1q, 2p, 3q, 5p, chr7, and 8q as well as arm level losses of 3p, chr4, 5q, 8p, 9p, 13p, and 17p have been identified. When comparing SQCC with ADC (Supplementary Fig. 1a), arm level gains of 2p, 3q and losses of 3p, chr4, 5q, and 13q were more prominent in SQCC. When comparing smokers versus non-smokers in ADC (Supplementary Fig. 1b), gains of 1q and chr2 are more prominent in smokers. Despite having a smaller magnitude of amplification and deletion frequencies relative to TCGA, CNVs in the CHOICE study were very consistent with those identified in TCGA dataset (Supplementary Fig. 2a–d). The focal CNV profile between the CHOICE and TCGA were compared to identify novel focal events in the Chinese population (Fig. 1). In both TCGA and CHOICE study, *SOX2*, *WHSC1L1-FGFR1*, *CCND1*, and *MYC* were identified to be the top focally amplified genes (Fig. 1a), while *CDKN2A*, *ERBB4*, *FAT1*, *PTPRD*, and *CSMD1* are among the top focally deleted genes in SQCC samples (Fig. 1b). For ADC, the top focally amplified genes are *NKX2-1*, *TERT*, *MDM2*, and *MCL1* genes (Fig. 1c), while significant focal deletions were observed for *CDKN2A* and *15q11.2* (containing *POTEB*, *NF1P2*, *SYK*, *RPL5*, and *PIK3R1*; Fig. 1d). Several focal events identified in the TCGA dataset including *9p21.1*, *LRP1B*, *FOXP1* in SQCC and *8q24.21*, *PTPRD*, *13q12.11* in ADC were not found in the current study. There was significant amplification and deletion events observed only in the CHOICE study but not in the TCGA dataset. Among all the CNVs that were unique to the CHOICE study, 15q11.2 was amplified in both SQCC and ADC, while 8p23.1 was amplified in ADC but deleted in SQCC. More interestingly, both amplification and deletion events were observed in 14q11.2 (containing *OR4K5*, *OR4K1, TRA, TRD*, and *CCNB1IP1)* in ADC, indicating a highly variable chromosome structure in this region.

### Somatic mutation and tumor mutation burden

An average of 8.0 somatic mutations per Mb was identified in ADC samples (4.6 non-synonymous mutations per Mb) and 11.8 somatic mutations per Mb (7.1 non-synonymous mutations per Mb) in the SQCC cohort. Mutation load has been identified as a strong predictor for immune checkpoint therapy^9^. In patients with ADC, smokers have higher mutation burden than non-smokers (Supplementary Fig. 3a).

**Fig. 2:**
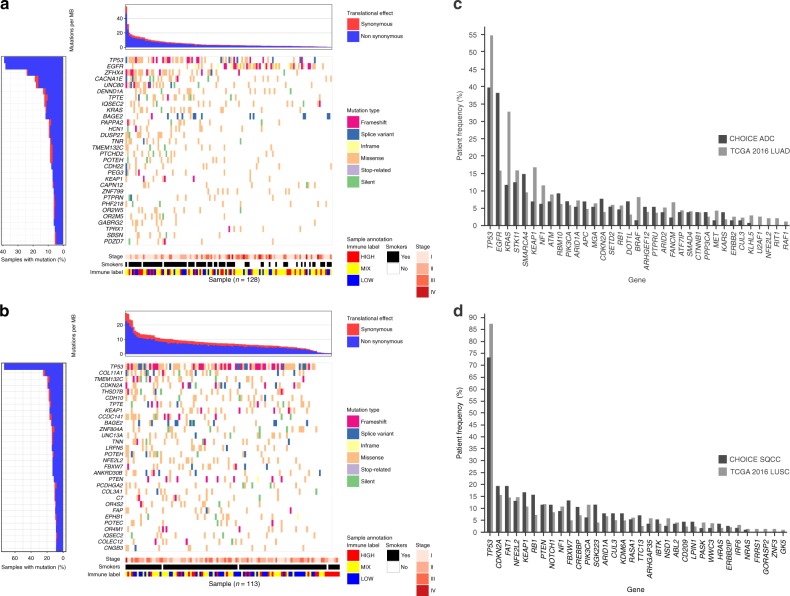
Significantly mutated genes for ADC and SQCC in the CHOICE study. **a** Top 30 significantly mutated genes in ADC, samples were ordered based on their somatic non-synonymous mutation burden (top panel) and genes were ranked by mutation frequencies (left panel). Cancer stage, smoker status, and immune infiltration status are annotated in the bottom panel. **b** Top 30 significantly mutated genes in SQCC. Comparison of the mutation frequencies of significantly mutated gene between the CHOICE and TCGA study in ADC (**c**) and SQCC (**d**). Source data are provided as a Source Data file

In lung ADC, *TP53*, *EGFR*, and *KRAS* were among the top mutated genes, whereas *TP53*, *KEAP1*, *NFE2L2*, and *KRTAP4-7* were the most significantly mutated (Fig. 2a, b, Supplementary Data 3 and 4) genes in SQCC. Compared to TCGA, *EGFR* was found to have higher frequency of somatic mutations in the CHOICE ADC patients (Fig. 2, 38.3% vs. 14.0%, Fisher’s exact test *p* = 5.3e-5*)*, whereas *KRAS* (10.9% vs. 33.0%, Fisher’s exact test *p* = 5.3e-5) and *BRAF* (0.8% vs. 10.0%, Fisher’s exact test *p* = 0.11) mutation frequencies were notably lower (Fig. 2c, Supplementary Fig. 4a, b; see Supplementary Data 5). Prominent ADC cancer-related genes including *TP53*, *KEAP1*, and *NF1* had lower somatic mutation rates, whereas *STK11, PIK3CA*, and *ARID1A* had consistent mutation rates between the two population (Fig. 2c, Supplementary Fig. 4a, b, Supplementary Data 5). Among non-smokers, the rate of *EGFR* mutation in ADC was elevated among women compared with men (75% [*n* = 28/37] vs. 50% [*n* = 5/10], Fisher’s exact test *p* = 0.14). For genes commonly altered in SQCC population, *TP53*, *RB1*, and *FBXW7* exhibited higher mutation rates, whereas *PIK3CA* had lower mutation rates in the CHOICE study (Fig. 2d, Supplementary Fig. 4c, and Supplementary Data 6). The mutation frequencies of *CDKN2A*, *FAT1*, *NFE2L2*, *KEAP1*, *PTEN*, *NF1, NOTCH1*, and *ARID1A* were comparable with the TCGA data. Higher mutation frequencies of *PABPC3* (14.2% vs. 2.7%, Fisher’s exact test *p* = 0.008) in SQCC and *IQSEC2* (10.9% vs. 2.3%, Fisher’s exact test *p* = 0.012) in ADC were observed in the CHOICE cohort.

The relationship between the cancer driver mutation and mutation load was examined and found that ADC patients with *EGFR* mutation or *ALK* fusion had significantly lower levels of mutation burden when compared with *EGFR* and *ALK* wild-type population. However, the same trend was not observed for patients harboring *KRAS* mutation (Supplementary Fig. 3b).

Mutation signatures were generated using a proportion of the nucleotide somatic alteration types^10^. Consistent with a previous report^11^, the CHOICE study exhibited an enrichment of the C > A mutation in the smokers compared with the non-smokers (Supplementary Fig. 5). No association was found in the nucleotide signatures between ADC and SQCC.

### Fusion detection

Canonical fusion genes detected in the CHOICE study included *ALK-EML4* (*n* = 5), *CCDC6-RET* (*n* = 1), *CD74-ROS1* (*n* = 1), *KIF5B-RET* (*n* = 1), and *FGFR3-TACC3* (*n* = 1) (Supplementary Data 7). All fusions were found in ADC except for a *FGFR3-TACC3* fusion which was found in SQCC. For all the fusion genes, the *ALK-EML4* fusion was the most frequent canonical fusion identified (4.2%, all in ADC), which is consistent with the general estimation of 4–7% in both the Western^12^ and Chinese population^13^. *ALK* rearrangements have historically been associated with non-smokers^14^ but the CHOICE study reported a larger proportion of *ALK-EML4* rearrangements from smokers (*n* = 3/5, 60%). This finding is consistent with a recent study in a large cohort of Chinese patients^15^ which found no association between smoking status and *ALK* rearrangements and may reflect a different demographic relationship compared with the Western population. In addition to the canonical fusions, there were several candidate recurrent novel gene fusions detected such as *CLTC-VMP1* and *PPFIBP1-STK38L*, which ranked high (>9) in the fusion scoring algorithm (see Supplementary Methods).

### Tumor-infiltrating lymphocyte enrichment profile in NSCLC

**Fig. 3:**
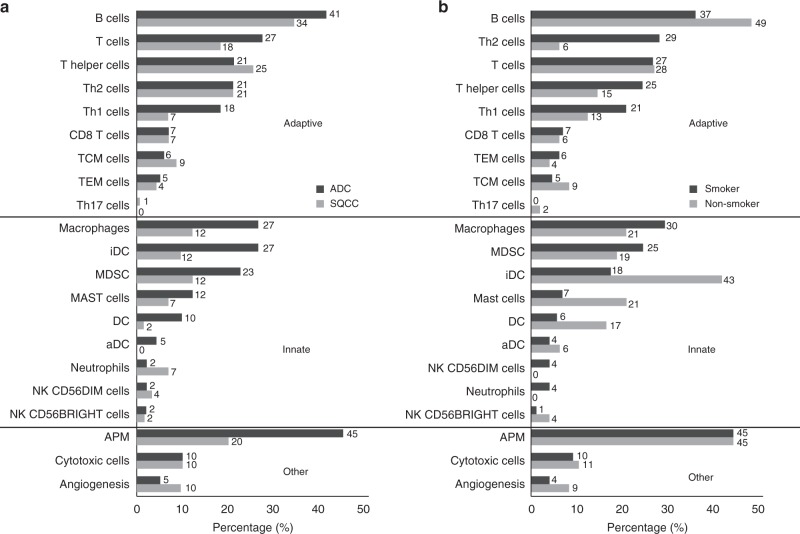
Tumor-infiltrating lymphocytes enrichment profiles. **a** Comparison of TILs enrichment profile in ADC (*n* = 131) versus SQCC (*n* = 114). **b** Comparison of TILs enrichment profile in smokers (*n* = 84) versus non-smokers (*n* = 47) in ADC. Percentage of patients with enriched immune cell signatures were calculated using ssGSEA (see Supplementary Methods). For each immune cell signature, enrichment is defined as *q*-value ≤0.1. Source data are provided as a Source Data file. **a** black bars indicate the percentage of patients having significant enrichment for the given immune cell type in ADC subtype, while gray bars represent the percentage in SQCC; **b** black bars represent the percentage of smokers, while gray bars represent the percentage of non-smokers. Immune cell signatures were classified to adaptive, innate and other

Enrichment of gene signatures for different immune cell types in adaptive and innate immunity, as well as gene signatures for antigen presenting machinery (APM), cytotoxic cell and angiogenesis were examined using single-sample Gene Set Enrichment Analysis (ssGSEA). Tumor-infiltrating lymphocytes (TILs) were shown to be significant in 72.5% of ADC (*n* = 95/131) and 54.4% of SQCC (*n* = 62/114; Fig. 3a). In the CHOICE cohort, effector memory T (TEM) cells, Tregs and natural killer (NK) cells were relatively low in both ADC (TEM: 5%, Treg: 0%, NK: 2%) and SQCC (TEM: 4%, Treg: 0%, NK: 0%). A higher percentage of APM (ADC 45% vs. SQCC 20%) and Th1 cells (ADC 18% vs. SQCC 7%) were observed in ADC, while an increased representation of B cell infiltration in both SQCC (41%) and ADC (34%) were also observed.

Between smokers and non-smokers, a similar level of TIL and APM enrichment was also observed (smokers 70.2% [*n* = 59/84] and non-smokers 76.6% [*n* = 36/47]), Fig. 3b). More smokers were estimated to have Th1, Th2, and macrophages in the TME, which may indicate inflammation response. Dendritic cells and B cells were shown to have a higher level in the TME of the non-smoker patients.

### Association between gene alteration and immune infiltration

**Fig. 4:**
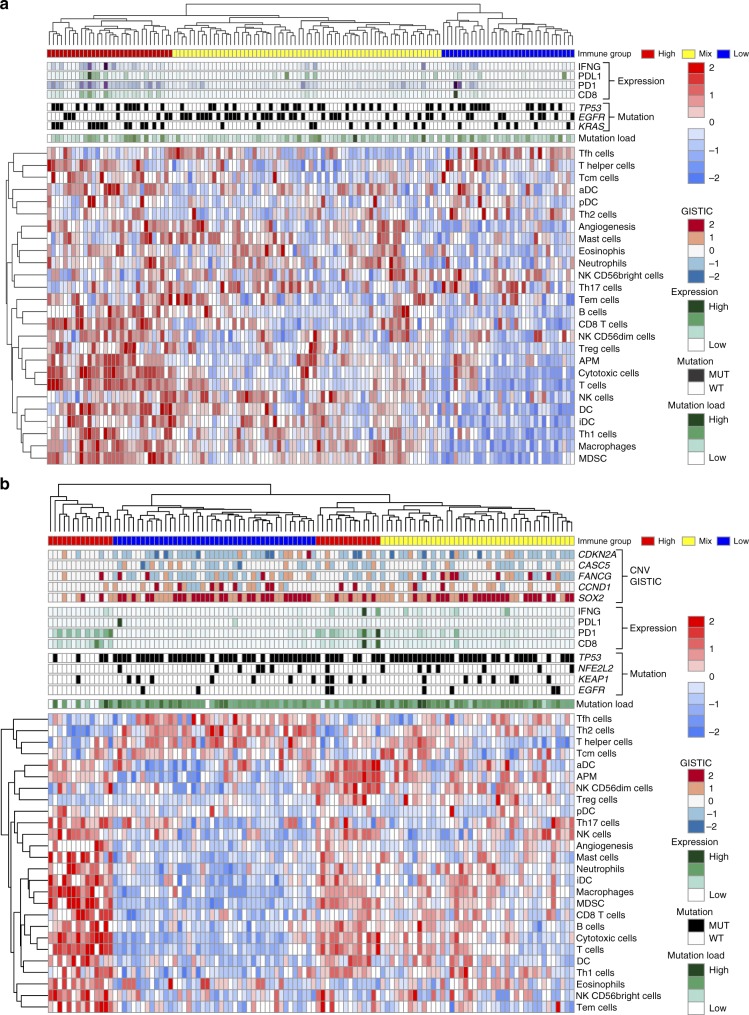
Characterization of immune infiltration in the CHOICE population. **a** Heat map of normalized ssGSEA score for ADC, mutation status of *TP53, KRAS*, and *EGFR* are shown while CNV level of *CASC5, FANCG, CDKN2A, CCND1*, and *SOX2* are shown. **b** Heat map of normalized ssGSEA for SQCC. Mutation status of *TP53, NFE2L2, KEAP1*, and *EGFR* are shown, while CNV profile of *CDKN2A, CASC5, FANCG, CCND1*, and *SOX2* are shown. Tumor samples were classified into 3 immune status: HIGH, MIX, and LOW based on the signature score of 26 immune cell types. Samples were also labeled using 5 types of omics data. (1) Mutation burden for each sample (green). (2) Immune status (red, yellow, and blue for HIGH, MIX, and LOW). (3) Selected significantly mutated genes in each subtype (black for mutation and white for wild-type). (4) mRNA expression value for four immune marker genes (*IFNG, PDL1, PD1,* and *CD8*) (dark green indicates high expression and light green indicate low expression). (5) GISTIC 2 based CNV for the selective gene. Source data are provided as a Source Data file. Dark red color represents homozygous amplification, light red for heterozygous amplification, white for diploid, light blue for heterozygous deletion, and dark blue for homozygous deletion

**Fig. 5:**
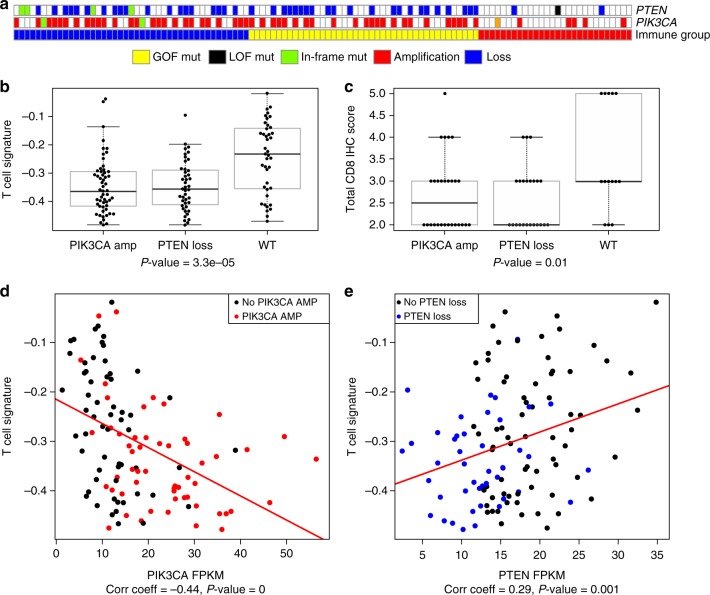
PI3K pathway activation versus T cell infiltration. **a** *PTEN* loss and *PIK3CA* amplification were enriched in immune LOW and MIX group SQCC (immune group red, yellow, and blue for HIGH, MIX, and LOW). **b** Comparison of *PTEN* and *PIK3CA* alteration with T cell signature. **c** Comparison of *PTEN* and *PIK3CA* alteration with CD8 IHC H-score. **d** *PIK3CA* expression versus T cell signature, amplification/high expression (red) was associated with low T cell signature. The top and bottom of the boxes are the lower and upper quartiles. The middle line in the box is median, and the whiskers are lowest and highest point within 1.5 times the interquartile range of the lower and upper quartile. **e** *PTEN* expression versus T cell signature, loss/low expression (blue) was associated with low T cell expression. Source data are provided as a Source Data file

The relative level of immune infiltration for each patient was investigated for ADC and SQCC. The ssGSEA based signature score of 26 immune cell types were used to cluster ADC (Fig. 4a) and SQCC (Fig. 4b) into 3 immune status (online methods): HIGH, patients having high ssGSEA scores of the various immune cell types; MIX, a mixture of high and low ssGSEA scores of the 26 immune cell types; and LOW, low ssGSEA scores of the 26 immune cell types. As expected, in both ADC and SQCC, patients with high immune marker gene expression values (*IFNG, PD-L1, PD-1,* and *CD8*) were enriched in immune HIGH population (Fig. 4a, b). Mutation load was not significantly associated with different immune status in both ADC and SQCC patient cohorts. When looking at mutation/amplification difference in ADC, immune HIGH tumors showed enrichment of *KRAS* mutation/amplification compared with the rest of ADC patients (immune MIX and LOW, Fisher’s exact test *p* = 0.0002). An opposite trend was observed for *EGFR* mutation (Fisher’s exact test *p* = 0.015, Supplementary Fig. 6a). *STK11* mutations were found to be associated with the relatively low level of PD-1, PD-L1, T-cells signatures and elevated level of neutrophils signature. However, the correlation is not significant except for T-cells (*t*-test *p* = 0.01, Supplementary Fig. 7). It has been reported that *STK11* deficiency can promote neutrophil recruitment and suppress T cell functions through the production of pro-inflammatory cytokines^16^. While in SQCC, immune MIX and LOW groups showed enrichment for amplification of *TP63*, *PIK3CA*, and *SOX2* as well as *TP53* and *NFE2L2* mutation (Supplementary Fig. 6b). *PTEN* loss and *PIK3CA* amplification are enriched in immune LOW and MIX group in SQCC patients (Fig. 5a). T cell signature and CD8 IHC score were found to be significantly reduced in those SQCC samples harboring *PTEN* loss or *PIK3CA* amplification (Fig. 5b, c). Furthermore, the high PIK3CA and/or low *PTEN* expression is significantly correlated with low T cell signatures in the SQCC samples (Fig. 5d, e). Loss of *PTEN* has been shown to increase the expression of immunosuppressive cytokines such as VEGFA and lead to reduced T cell infiltration and response to T cell-mediated immunotherapy in melanoma^17^.

Expression profiles were also examined for differently expressed genes among the three immune groups. In total, 147 genes in ADC and 437 genes in SQCC were identified to be significantly differentially expressed among three immune groups (Supplementary Fig. 8a, b). Pathway enrichment analysis identified enrichment of immune-related pathways in ADC (Supplementary Fig. 8c), while in SQCC, top-ranked enrichment pathways were driven by different classical cancer-related signal transduction pathways (Supplementary Fig. 8d). The pathway analysis identified genes in WNT pathway that are also differentially expressed. It is consistent with recent findings that activated WNT/β-catenin pathway is associated with TME immune cell exclusion in melanoma^18^. The activated WNT pathway can down-regulate CCL4 expression via ATF3-dependent transcriptional repression, which will lead to reduced infiltration and activation of dendritic cells and CD8+T cells^18^. Several cytokines including CXCR3, CXCL9, CXCL10, IL2, and VEGFA were identified to be differentially expressed between immune groups (Supplementary Fig. 9a), which suggest immune exclusion through lack of innate immune sensing or lack of effector T cell recruitment^19^. WNT pathway activity was estimated by ssGSEA using WNT pathway hallmark gene set from MSigDB. The WNT pathway signature were shown to be significantly negatively correlated with both CCL4 mRNA expression and T cell signatures (Supplementary Fig. 9b).

### Immunohistochemistry staining

The PD-L1 expression on the tumor cell and in the tumor microenvironment

**Fig. 6:**
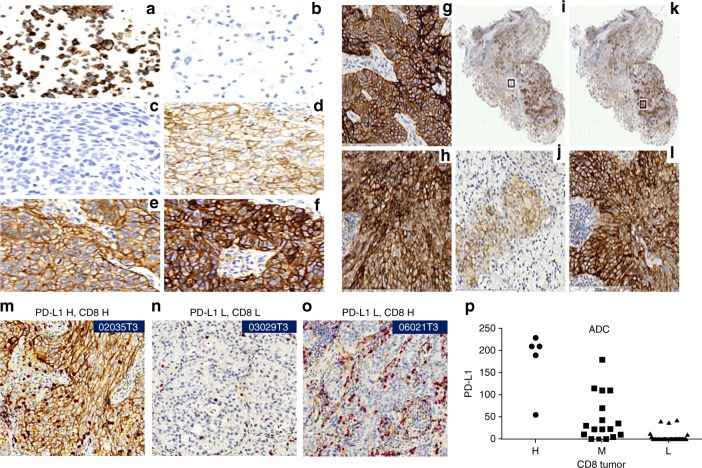
Immunohistochemistry staining of PD-L1 and CD8. **a** Membrane staining (3+) was seen in positive control cells (HDLM-2). **b** No staining was seen in negative control cells (PC3). **c**–**f** Representative images of membrane staining on tumor cells were shown with different signal intensities: **c** No staining. **d** Low/1+staining, **e** Medium/2+staining. **f** High/3+staining. Example of heterogeneous IHC staining: **g** Strong staining of PD-L1 from SQCC (**h**) Strong staining in ADC. **i**–**l** Heterogeneous expression of PD-L1 from the same tissue. **i**, **k** are the same tissue; two regions are selected (boxed). **j** Zoomed-in region of **i**. **l** Zoomed-in region of **k**. Correlation between PD-L1 and CD8. **m**–**o** Representative images of PD-L1/CD8 both high, both low and PD-L1 L/CD8 H. **p** Correlation between CD8 positivity and PD-L1 expression was observed in ADC (ANOVA *p*-value = 8.76e-13, *F* = 50.25, DF = 2). Source data are provided as a Source Data file

To characterize tissue expression of PD-L1 (PD-L1 rabbit mAb, 0.7 mg/mL, cat. no: 13684, cell signaling, diluted at 1:100) in Chinese NSCLC, formalin-fixed paraffin-embedded (FFPE) sections from 147 cases were subjected to immunohistochemistry (IHC) analysis. The PD-L1 positivity rate in the CHOICE study was 23.1% using H-score ≥50, or 63.9% using >1% tumor cell positive as a cutoff, which is consistent with what was reported in the literature on the Western population (Fig. 6a–f). Both PD-L1 protein and mRNA expression were found to be significantly higher in ADC smokers (Supplementary Fig. 3c). Consistent with temporal and spatial expression of PD-L1 from previous reports^20,21^, expression of PD-L1 showed inter- and intra-tumor heterogeneity in both ADC and SQCC (Fig. 6g–l). This heterogeneity could contribute to challenges of utilizing PD-L1 expression as companion diagnostic markers for predicting the response from anti-PD therapies, especially by testing on small biopsy samples.

### Correlation of PD-L1 and CD8

To evaluate the expression of PD-L1 and CD8 in Chinese NSCLC, dual-color IHC method was developed and applied to the CHOICE cohort. PD-L1 expression was quantified in the same way as previously described. CD8 expression in tumor and stroma regions was semi-quantified and categorized into H, M, and L classes. Like PD-L1 expression, CD8 positivity also showed inter- and intra-tumor heterogeneity. A strong correlation was observed for the PD-L1 and CD8 protein expression in ADC (ANOVA *p*-value = 8.76e-13), but not in SQCC (Fig. 6m–p). In addition, T cell signature was also shown to be significantly correlating with PDL1 mRNA expression (Supplementary Fig. 10).

### Survival analysis of immune group and immune signature

**Fig. 7:**
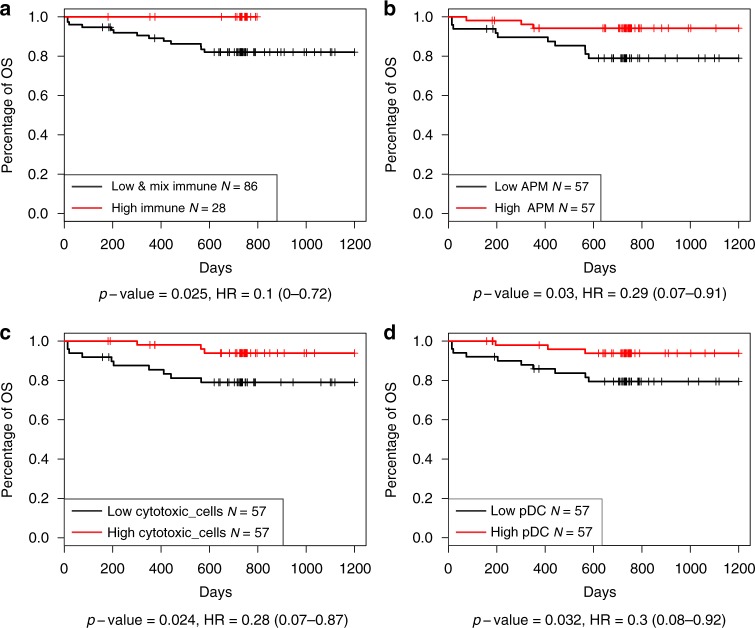
Immune signatures associated with SQCC patient overall. **a** Immune signature, immune MIX and LOW groups were combined together and compared with the immune HIGH group. **b** APM signature. **c** Cytotoxic cell signature. **d** pDC signature. Source data are provided as a Source Data file

The patients in the CHOICE study were followed-up for at least 2 years. To verify the validity of survival data, the relationship of known factors such as stage and smoking status with patient outcome were tested. High tumor stage was found to be significantly associated with patients’ overall survival (OS) time in both ADC and SQCC (Supplementary Fig. 11). Smokers were found to have shorter PFS time in ADC cohort. The prognostic role of the immune cell signatures, the three immune cluster groups, and mutational load were evaluated. The immune HIGH group was shown to have favorable OS time when compared with immune LOW and MIX group in SQCC patients in the Kaplan–Meier survival analysis (Fig. 7a). The survival analysis of immune signature showed that only Tfh cells signature (log-rank test *p* = 0.01) was found to be significantly associated with patient OS in ADC patients. Higher APM (log-rank test *p* = 0.03), cytotoxic T cell (log-rank test *p* = 0.02), and pDC (log-rank test *p* = 0.03) signature was associated with longer OS survival in patients with SQCC (Fig. 7b, c, Supplementary Data 9). The multivariate analysis which take into account of age, stage, and treatment methods showed that the APM and cytotoxic T cell signature remained significant (Cox regression, *p*-value ≤0.01), but not significant for the immune group.

## Discussion

The CHOICE study performed comprehensive molecular analysis of the CNV, SNV, and transcriptomic profiles in Chinese NSCLC patients and compared the results with the TCGA NSCLC dataset that mainly composed of the Western population. Higher frequency of 8p23.1 loss was found to be significant in the CHOICE study. Genes in this region, such as *MSRA*, *MFHAS1*, *DEFB106A*, and *DEFB105A*, play an important role in the initiation and progression of lung SQCC^22^. Focal alteration at 14q11.2 containing olfactory receptor family genes was specifically identified in lung ADC. It has been reported that smoking is associated with decreased olfactory performance^23^. However, little is known about the functional and integrative mechanisms of the human olfactory receptor in odorant perception in lung cancer. Focal deletions of *LRP1B*, a putative tumor suppressor in lung cancer was not found in the CHOICE study (Fig. 1) but in TCGA. Focal amplifications at 8q24.21 were unique to TCGA, which contain *MYC* and *MIR1208*. It has been suggested that they could be used as biomarkers to select patients with poor prognosis in lung ADC^24^.

In lung ADC, the *EGFR* somatic mutation frequency in the CHOICE study was notably higher (38.3%) compared with the rate typically found in the Western population (14–21%)^4,25^. Interestingly, a higher rate of *EGFR* mutation was observed among women ADC non-smokers (75%) compared with previous studies^26,27^. While it was clear that histology and smoking status are associated with *EGFR* status, the effect of gender remains unclear in Asian population^26,28^, partly due to the high rate of non-smokers in this women population. Unfortunately, the CHOICE study, with the limited number of male non-smokers does not shed any additional light on the effect of gender in this regard. Lower somatic mutation frequencies were observed for *KRAS* (10.9%) and *BRAF* (0.8%) compared with the Western population (33–38% for *KRAS* and 2–10% for *BRAF*)^4,29^, which are consistent with other studies^30,31^. *KEAP1*, *NFE2L2*, and *CUL3* are responsible for dysregulation of oxidative stress pathway in lung cancer and they have been shown to have elevated mutation rates in NSCLC. Compared to TCGA dataset, ~13% reduction of *KEAP1*/*NFE2L2*/*CUL3*) mutations in ADC (7.8% vs. 20%) was observed (Supplementary Data 5) and a 5% increase in SQCC (36.3% vs. 31%) (Supplementary Data 6) in the CHOICE study. This finding is interesting given the important role of *KEAP1* in antioxidant stress response in the context of elevated smoking frequencies found in Chinese NSCLC patients. The mutation rate is elevated in the CHOICE SQCC (13.3% vs. 5.2%). The identification of these somatic alterations and their variable frequencies should be interpreted with some caution as NGS data can be subject to numerous sources of technical variation including library prep, sequencing, computational analysis, etc.

It is important to understand the immunological landscape of NSCLC in the Chinese population, as this information might reveal the mechanism of response and resistance to specific immunomodulatory agents and inform future development of more effective combination approaches. For example, in addition to the PD-1/PD-L1 axis of immunosuppressive mechanism, the presence of MDSCs and Tregs could also contribute to additional immune suppression in NSCLC. The ssGSEA was used to characterize the relative composition of infiltrating immune cells in TME. The preliminary findings suggest a diversity in NSCLC TMEs, offering a comprehensive view of the relative level of enrichment of different immune subtypes and provided insights about the complex interactions of NSCLC tumor cells with its microenvironment. Specifically, the enrichment profile of TILs suggests the presence of innate immune cells such as macrophages, myeloid-derived suppressor cells (MDSCs), and dendritic cells.

Using ssGSEA based immune signature, enrichment of T cells, B cells, and a reduction of NK cells in NSCLC was demonstrated, which is consistent with a recent multiscale immune profiling study of stage I lung ADC lesions using Cytometry by Time-Of-Flight (CyTOF)^32^. While most studies have focused on the cell type with best anti-tumoral potential, such as CD8+T cells, not much is known regarding tumor-infiltrating B cells. There is recent evidence supporting a complex role for TIL-B cells in modulating the immune response across solid tumors^33^. B regulatory (Breg) cells express cytokines such as IL-10, TGF-β, and immune regulatory ligands such as PD-L1 have been shown to suppress T cell responses in tumors from different anatomical origins^33^. In lung cancer, it has been suggested that TIL-B cells help to generate potent, long-term immune responses against cancer by presenting tumor antigens to CD4 TILs^33^ and their presence is correlated with improved survival^34^.

Tumor-infiltrating B cells have been studied most extensively in breast cancer, where they are present in 25% of tumors and comprise up to 40% of the tumor-infiltrating lymphocytes and this has been associated with favorable survival rates in breast cancer^35,36^. Tumor-infiltrating B cells are also found in tertiary lymphoid structures consisting of CD4+, CD8+T, and dendritic cells, which could promote the formation of tertiary lymphoid structures by secreting lymphotoxin and chemokines that attract and stimulate T cells, dendritic cells, and other immune cells^37,38^. On the other hand, in mouse tumor model, B cells were shown to generally inhibit the T cell response. A key factor may be the activation status of B cells in different contexts. The T cell response was inhibited by resting B cells and facilitated by activated B cells. The enrichment profile of B cell infiltration suggests that there is an opportunity to target tumor cells through regulation of the B cell functions. Follow-up studies are warranted to confirm these findings, to better understand the function and types of B cells in the TME.

Despite promising clinical efficacy in patients with various cancers^39,40^, many patients with NSCLC fail to respond to anti-PD-1/PD-L1 treatment. The CHOICE study observed inter- and intra-tumor heterogeneity of PD-L1 expression (Fig. 6), which could contribute to challenges of utilizing PD-L1 expression as a companion diagnostic marker for predicting the response from anti-PD therapies. The CHOICE study also evaluated the expression of PD-1 and CD8 (Fig. 6m–p) and found a correlation in ADC but not in SQCC. Expression of PD-L1 has been hypothesized to be regulated by IFN-γ released by TILs^41,42^, thus spatial proximity between PD-L1 expression and T-cell is a consideration. PD-L1 mRNA expression was shown to be correlated with T cell signature (Spearman’s correlation coefficient = 0.57, Supplementary Fig. 10). This finding needs to be further confirmed to examine if the same biomarker strategy would be used for different histology subtypes.

In SQCC, one subpopulation of immune HIGH patients tends to have a lower mutation burden, suggesting that the specific type of neo-antigen might be the key factor to promote the immune response in TME. A recent study has reported that mutations in certain genes like *CASP8* or *TP53* may affect host immune response to tumors. Identification of such gene may lead to the development of personalized immune-based therapy^43^. Several mutations/alterations that might play important role in regulating the immune response were identified. Loss of *PTEN* and/or *PIK3CA* amplification, as well as the activation of WNT pathway in immune LOW samples in SQCC, were associated with reduced immune cell signals derived by using the immune signatures. These findings suggest that targeting PI3K or WNT pathway may potentially further enhance the efficacy of immunotherapy such as PD1 in SQCC.

With the biological and analytical limitations of PD-1/PD-L1 IHC, it is more likely to get a better predictive value when combining biomarkers from different perspectives. Recent publications suggest that multifactorial biomarkers may be needed to predict the response of anti-PD therapies, including PD-L1 expression, TILs, tumor antigens, and mutational load^40^. The CHOICE study generated data for genetic profiling, PD-L1 expression by IHC and TIL status, and provided comprehensive descriptive data to characterize these potential biomarkers in Chinese NSCLC population. Further analysis will be done to evaluate the correlation between PD-L1 expression and other biomarkers to find potential complementary markers for PD-L1 IHC, along with future follow-up data for patient outcome.

Finally, our analysis of the relative tumor immune composition has certain inherited limitations. First, given the heterogeneity of tumor and complexity of TME, the gene signatures can only suggest the relative presence of a given immune cell type but cannot be used to quantify the composition of the immune environment. Second, given our limited understanding of immune response in TME, the genes used to generate the immune signatures might need further optimization to better characterize immune cell types in NSCLC.

## Methods

### Sample collection

This study was conducted between September 2013 to October 2016 across 6 hospitals in China (Guangdong Provincial People’s Hospital, Shanghai Zhongshan Hospital, Peking University People’s Hospital, Nanjing Medical University Affiliated Cancer Hospital, 1st Hospital of Jilin University and Jilin Provincial Cancer Hospital; clinicaltrials.gov identifier: NCT02113852). All patients provided written informed consent to conduct genomic studies in accordance with the ethical principles laid down in the Declaration of Helsinki, the International Conference on Harmonization Guidelines for Good Clinical Practice, applicable regulatory requirements and Janssen’s policy on bioethics. The study was approved by the ethical committees of all participating hospitals. Samples were either collected prospectively from lung cancer surgical material or retrospectively (stage IA-IIIA) from the tumor Biobank between 2006 and 2012. Patients with a history of chemotherapy, biological, immunological therapy, or radical radiotherapy were excluded.

Demographic data, Eastern Cooperative Oncology Group performance status, concurrent medications, medical, surgical, and smoking history were obtained. Patients were categorized based on their smoking habits as smokers (individuals smoke >20 packs/year or <10 years of smoking cessation history prior to enrollment) and non-smokers (individuals smoke <100 cigarettes in their lifetime). Surgically resected tumors, adjacent normal tissue, and matched blood samples were collected and snap-frozen. Histopathological review of HE stained sections was performed by pathologists. Only tumor with tumor content ≥60% were selected for profiling analysis.

DNA from the frozen tissue was extracted from the using QIAamp DNA Mini Kit (51306) and QIAamp DNA Blood Kit (51106) was used for blood DNA. RNA isolation was performed using Trizol and RNeasy MinElute Cleanup Kit (74204).

### RNA sequencing for expression analysis

RNA quality was analyzed using Bioanalyzer 2100 and only samples with RIN >8 were used. RNA library was prepared using TruSeq RNA sample prep kit (RS-122-2001). RNA-seq data were obtained for both tumor and matched adjacent normal samples for each patient. RNASeq data were obtained for both tumor and matched adjacent normal samples for each patient. The raw reads were aligned to the Human genome GRCh37 Gencode v19 using STAR (v2.4.2a)^44^ with the following setting: outFilterMismatchNmax = 10, outFilterMismatchNoverReadLmax = 0.04, outSAMstrandField = “intronMotif”. Cufflinks (v2.2) was used with multiple read error correction option to generate FPKM data^45^.

### Whole-exon sequencing and mutation analysis

Whole-exome sequencing for tumor and matched blood from all patients were performed on Illumina Hiseq2000 using 2 × 100 bp pair-end sequencing method. The average data output was about 200×coverage. Tumor and normal sequencing FASTQ data were first aligned to the hg19 UCSC reference genome using Novoalign (v2.03) with the following options: using base quality sequence calibration (“-k”), Illumina adapter removal (“-a”), reduction of the alignment score threshold for low complexity reads (“--hlimit 8”), hard clipping of low-quality bases with a quality < 20 (“-H 20”), filtering of polyclonal reads (“-p 5,20”) and reduction of the alignment score threshold (“-p 7,20”). Then SAM files were converted to BAM format using samtools (v 0.1) and subsequently deduped and sorted using Novosort (v1.03.07). Base quality recalibration, indel realignment were performed using GATK (v1.6)^46^.

Variant calls were generated using a combination of multiple callers including GATK UnifiedGenotyper (v1.6)^46^, Somatic Sniper (v1.0.5.0)^47^ LowFreq (v2.1.2)^48^ Strelka (v1)^49^, and VarScan 2 (v2.3.5)^50^. The settings for the variant callers are: GATK UnifiedGenotyper (run on both tumor and normal samples, subtract normal variants from tumor, -glm BOTH, -dbsnp137); SomaticSniper (min alignment mapping quality (Q) > 10 min read depth = 20); Strelka (default setting); Varscan (min-coverage-tumor = 8, somatic-*p*-value = 0.99); LoFreq (“lofreq somatic” with default setting).

SNV and short indel calls generated from each tool were merged and then annotated using the UCSC hg19 database with SNPEff (v4.0)^51^. ExaC (r0.2), 1000 Genomes (phase 1), Mills, COSMIC (v74), and dbSNP 138 were also used for the variant annotation.

The somatic variant calls were generated with the following steps: (1) remove variants with less than 8 supporting reads and exclude any synonymous and UTR variants, (2) germline variants from the matched normal tissues were subtracted from each tumor variant results, (3) any variants identified by the 1000 genome project and ExAC were excluded (r0.2), (4) the pooled germline variants identified from 128 blood samples (57 ADC and 71 SQCC patients) were removed from the final variant calls. To assess the significance of the SNVs, all variants were processed by MutSigCV^52^. The TCGA data was obtained from 2016 PanCancer study in cBioPortal (www.cbioportal.org) using CGDSR R package^53,54^.

### Fusion detection

Reads (FASTQ) were aligned to the transcriptome using the STAR aligner^44^ and fusions were called using a combination of three fusion callers: defuse, TopHat-fusion, and FusionCatcher. Fusion candidates were then compiled and scored based on key characteristics consistent with what has been observed in canonical fusions. These features include (i) count of junction and mate pair reads, (ii) presence of one or more genes in canonical fusions described in the literature^55^, (iii) number of fusion callers detecting the fusion, (iv) presence of either partner in the cancer census^56^, and (v) the recurrence of fusions found in this dataset.

### Somatic copy number data generation and data analysis

Total 238 tumor DNA samples (ADC: 124 and SQCC: 114) were hybridized to the Affymetrix SNP 6.0 array. Copy number variations of tumor samples were analyzed using the 17 blood samples as a pooled normal reference and the results were delivered as copy number status of each individual gene. Data were subsequently processed from the raw CEL files using Affymetrix Genotyping Console 4.2 to infer a preliminary copy-number at each probe locus. For each tumor sample, log ratios were computed by normalizing against a modified reference derived from the 17 normal samples and median normalization was then used to normalize all of the tumor samples to the median value of the individual tumor sample. The Bioconductor DNA Copy package was used to analyze SNP6 log-ratio calls for all tumor samples in ADC and SQCC. Circular Binary Segmentation SEG values were generated with adjusted *p* values using standard workflow recommended for CNV detection. Significant focal copy number alterations were identified from segmented data using GISTIC 2.0 according to the publisher’s recommendations for cancer cohorts. Two peaks were considered similar across different data sets if the known target gene of each peak was the same, or they were located within the same cytoband (the genomic locations of the peaks overlapped after adding 1 Mb to the start and end locations of each gene).

### IHC staining and image analysis

Tumor samples were obtained from 147 surgical resections and processed to make FFPE blocks following standard procedure. Prior to IHC staining, 4 µm serial sections were prepared for IHC staining of PD-L1 and cluster of differentiation 8 (CD8). Dual color IHC of PD-1 and CD8, as well as hematoxylin and eosin staining, were also performed. All stained slides were evaluated in a blinded fashion by one scientist trained to identify the features of NSCLC and scoring via criteria defined by a pathologist. Slides were examined for the presence of PD-L1 and CD8 within the tumor nest and the stroma. Correlation between PD-L1 and CD8 expression were analyzed semi-quantitatively. CD8 expression in tumor and stroma regions were semi-quantified and categorized into H, M, and L classes. Positivity of PD-L1 was defined using various criteria, such as H-score ≥50%, tumor cell positive >50% and tumor cell positive >1%.

### Tumor-infiltrating immune cell analysis

Marker genes for immune cell types and angiogenesis marker genes were identified^57,58^. Of 26 gene signatures, 11 were for immune cells in adaptive immunity, 12 for innate immunity, and 3 for MDSC, angiogenesis, and antigen presentation machinery. Tumor RNA from 131 ADC and 114 SQCC lung samples were used in the analysis (Supplementary Data 8). The immune signature score was calculated using ssGSEA method implemented by R package GSVA^59^. The tumor-infiltrating lymphocytes enrichment profiles was generated using the Preranked GSEA method^60^. Briefly, the RNAseq data was first *z*-score normalized. Then the gene *z*-score were ranked for each patient and the immune signature gene lists were selected for comparison of this ranking using the GSEA method. The immune signature gene lists with false discovery rate (*q*-value) <10% were considered as over-represented or enriched.

The immune groups were explored using hierarchical clustering in combination of considering T cell and B cell immune signature levels. There are two major clusters identified in the ADC immune signature data (relatively high and low). Within the cluster with relatively high immune signatures, the patient samples can be further divided into two subgroups (high and mix group) based on T cell, and B cell signature levels. For SCC samples, hierarchical clustering identified three major clusters corresponding to high, low, and mix immune groups. Within the mix group, a subset of samples show enrichment in B cells, T cells, and elevated PD1 and CD8 expression. This group of patients were classified as immune high subtype.

To identify differentially expressed genes among three immune groups, ANOVA and Kruskal–Wallis tests were used. Only genes with FDR <0.01 for both tests were selected. To minimize overfit, those genes were later filtered to make sure their expression was not correlated (correlation coefficient <0.6) with any immune markers used to classify the patients. At last, genes were further filtered by comparing their mean expression values among three different immune groups. A fold-change bigger than 2 must be observed from 3 possible pair-wise combinations. Metacore (V6.31) curated Pathway Maps database was used for the enrichment analysis and FDR was used to adjust for multiple testing.

### Mutation landscape figure generation

The waterfall plot of a mutational landscape was generated using GenVisR package^61^. Mutation types were retrieved from MutSigCV2 and were ordered by its potential impact from most deleterious to least. Mutation burden values were also calculated from MutSigCV2 results.

### Statistical analysis

All statistical analysis were performed in R. *t*-test was used for two group comparison and ANOVA or Kruskal–Wallis test was used for more than two group comparisons. Multiple comparison corrections were used to calculate q-values using Benjamini-Hochberg method. Fisher’s exact test was used to compare two categorical variables. For survival analysis, patient’s survival data was right-censored at 1200 days. Survival analysis was performed using Kaplan–Meier survival plot and log-rank test *p* value was calculated. Cox regression was used in the multivariate analysis to adjust for age, stage and treatment effects. Firth’s penalized maximum likelihood bias reduction method was implemented using coxphf package to calculate the hazard ratio and 95% confidence intervals.

### Reporting summary

Further information on research design is available in the Nature Research Reporting Summary linked to this article.

## Supplementary information

Supplementary Information

Description of Additional Supplementary Files

Supplementary Data 1

Supplementary Data 2

Supplementary Data 3

Supplementary Data 4

Supplementary Data 5

Supplementary Data 6

Supplementary Data 7

Supplementary Data 8

Supplementary Data 9

Supplementary Data 10

Supplementary Data 11

Reporting Summary

Source Data

## Data Availability

Patient clinical data (deidentified) were provided in the Supplementary Data 1. The complete somatic mutation calls can be found in Supplementary Data 10 and 11. The VCF files of Exome-seq data have been deposited to the European Variation Archive (EVA) at the EMBL-EBI under accession number PRJEB31315 (https://www.ebi.ac.uk/eva/?eva-study=PRJEB31315). The RNA-seq FPKM data have been deposited at figshare (10.6084/m9.figshare.7306364.v1). The source data underlying Figs. 1, 2c–d, 3–5, 6p, and 7 and Supplementary Figs. 2, 3, 9, and 10 are provided as Source Data file. All other relevant data are available from the authors of this study upon request.
