# Supplementary material for: Comprehensive genomic and immunological characterization of Chinese non-small cell lung cancer patients
Source: Nat Commun. 2019 Apr 16;10:1772. doi: 10.1038/s41467-019-09762-1 (PMC6467893; doi:10.1038/s41467-019-09762-1)
Supplement: Supplementary file 1 — Supplementary Information [file 41467_2019_9762_MOESM1_ESM.docx]

# SUPPLEMENTARY INFORMATION

# Comprehensive Genomic and Immunological Characterization of Chinese Non-Small Cell Lung Cancer Patients

Zhang et al.

**
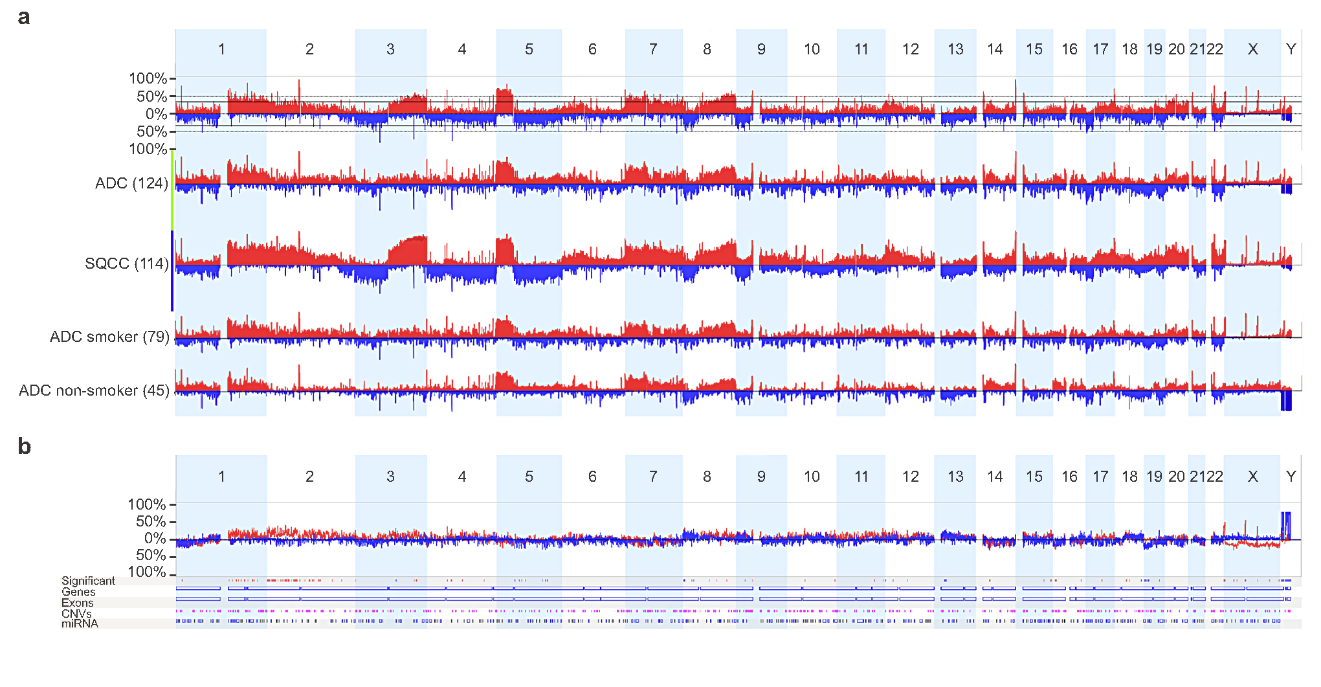
**

**Supplementary Figure 1. Overview of genomic copy number alterations in the CHOICE population. Arm level CNVs were plotted for 238 CHOICE samples with CNV data for comparison. (a) Comparison between ADC (124) and SQCC (114). (b) Comparison between smokers (79) and non-smokers (45) in ADC. Copy number gain is indicated in red and copy number loss is indicated in blue.**

**
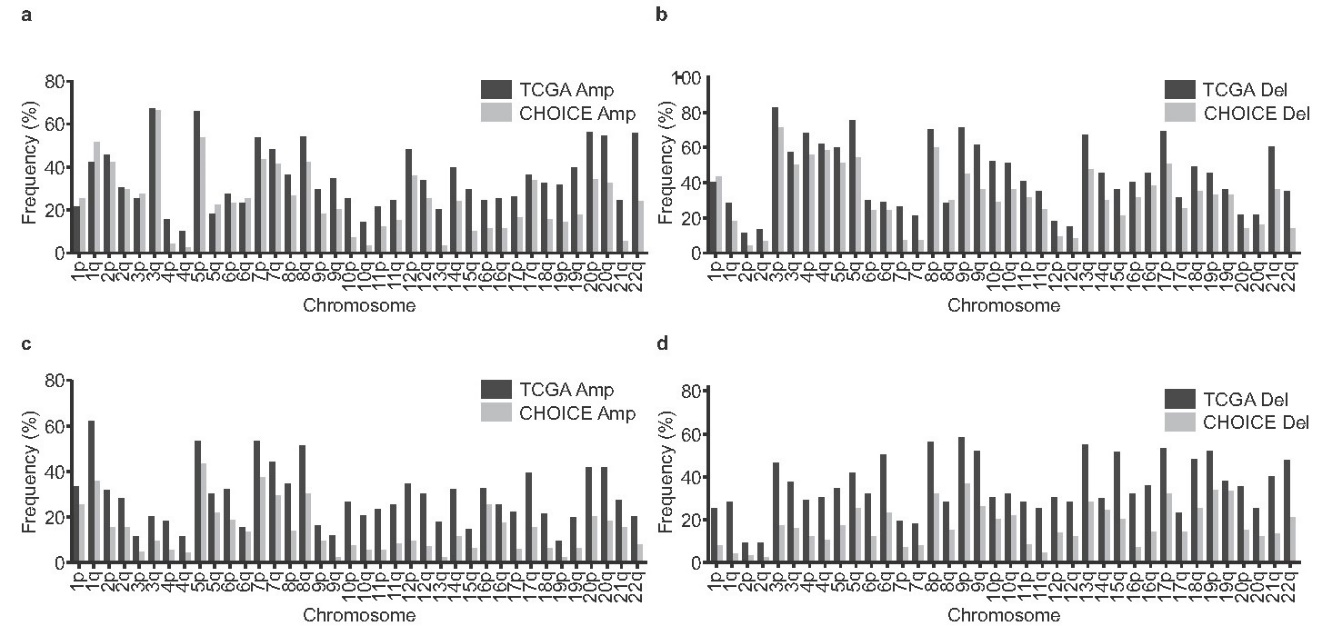
**

**Supplementary Figure 2. Comparison of arm level copy number alterations between CHOICE and TCGA datasets. (a) Amplification frequencies of CHOICE versus TCGA in SQCC. (b) Deletion frequencies of CHOICE versus TCGA in SQCC. (c) Amplification frequencies of CHOICE versus TCGA in ADC. (d) Deletion frequencies of CHOICE versus TCGA in ADC. The GISTIC 2.0 algorithm was used to identify amplification and deletion events to calculate frequencies at chromosome arm level.** Source data are provided as a Source Data file**.**

**
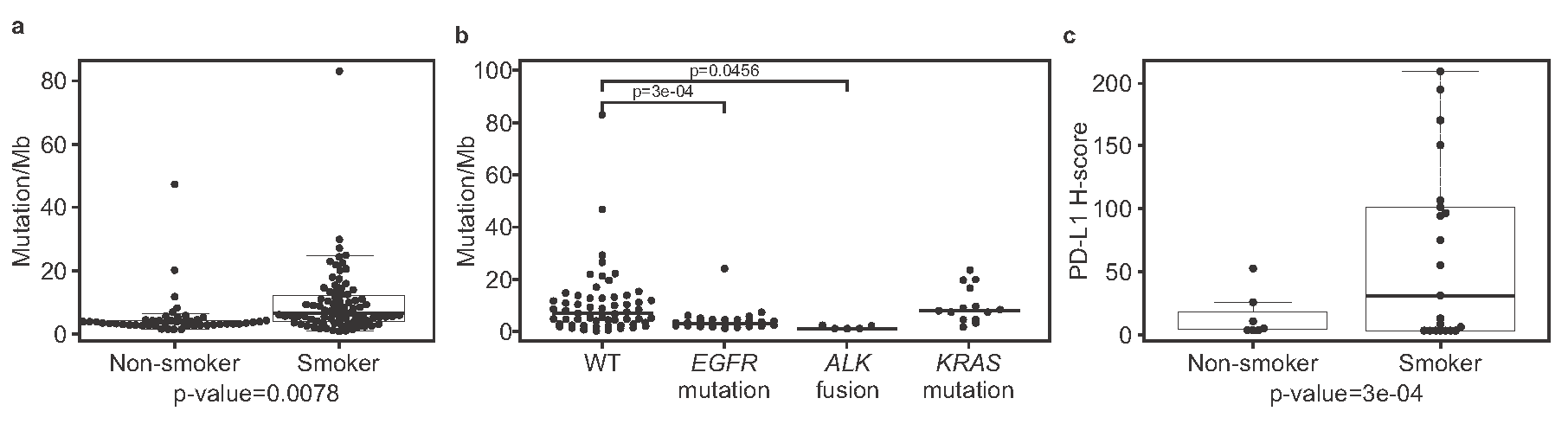
Supplementary Figure 3. Mutation load and smoking status in ADC patients. The relationship among cancer driver mutation, mutation load/burden as well as smoking status were compared in CHOICE ADC patients. (a) Driver mutation versus mutation load. (b) Difference in mutation load/burden between smoker and non-smoker. (c) PD-L1 IHC H-score versus smoking status. The top and bottom of the boxes are the lower and upper quartiles. The middle line in the box is median, and the whiskers are lowest and highest point within 1.5 times the interquartile range of the lower and upper quartile.** Source data are provided as a Source Data file**.**

**
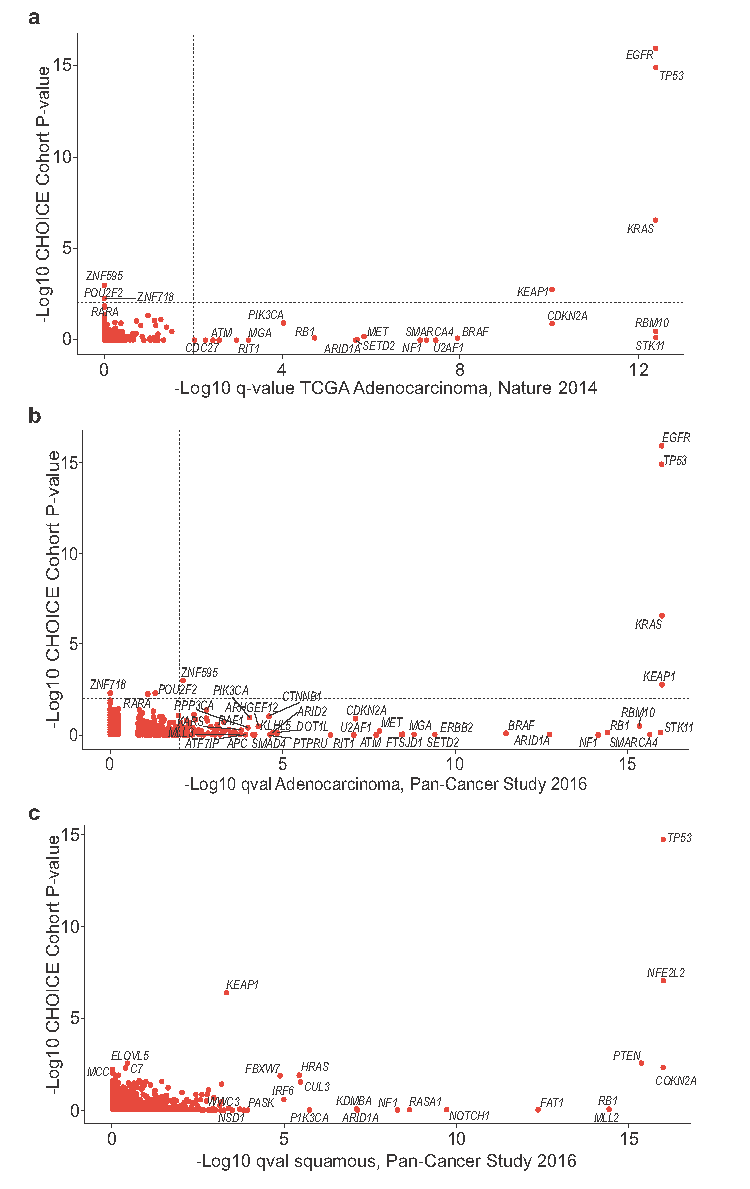
**

**Supplementary Figure 4. Mutation data comparing with different TCGA dataset. (a) CHOICE ADC versus TCGA cohort 2014. (b) CHOICE ADC versus Pan cancer cohort 2016. (c) CHOICE SQCC versus Pan cancer cohort 2016. -Log10 based p value for CHOICE study and -log10 based q value from different TCGA cohort were plotted. p value of 0.001 (horizontal dashed line) and q-value of 0.01 (vertical line) were used as cutoff for statistical significant, data point above and on the right of the dashed lines were considered significantly mutated genes in either of both cohort. Due to smaller sample size, CHOICE cohort has less significant mutated genes compared with the TCGA dataset. TCGA publication for SQCC in 2012 does not provide any q value for mutation calls, thus it is not included in the plot. Q-values for CHOICE study are available in Supplementary Table 3 and 4.**

**
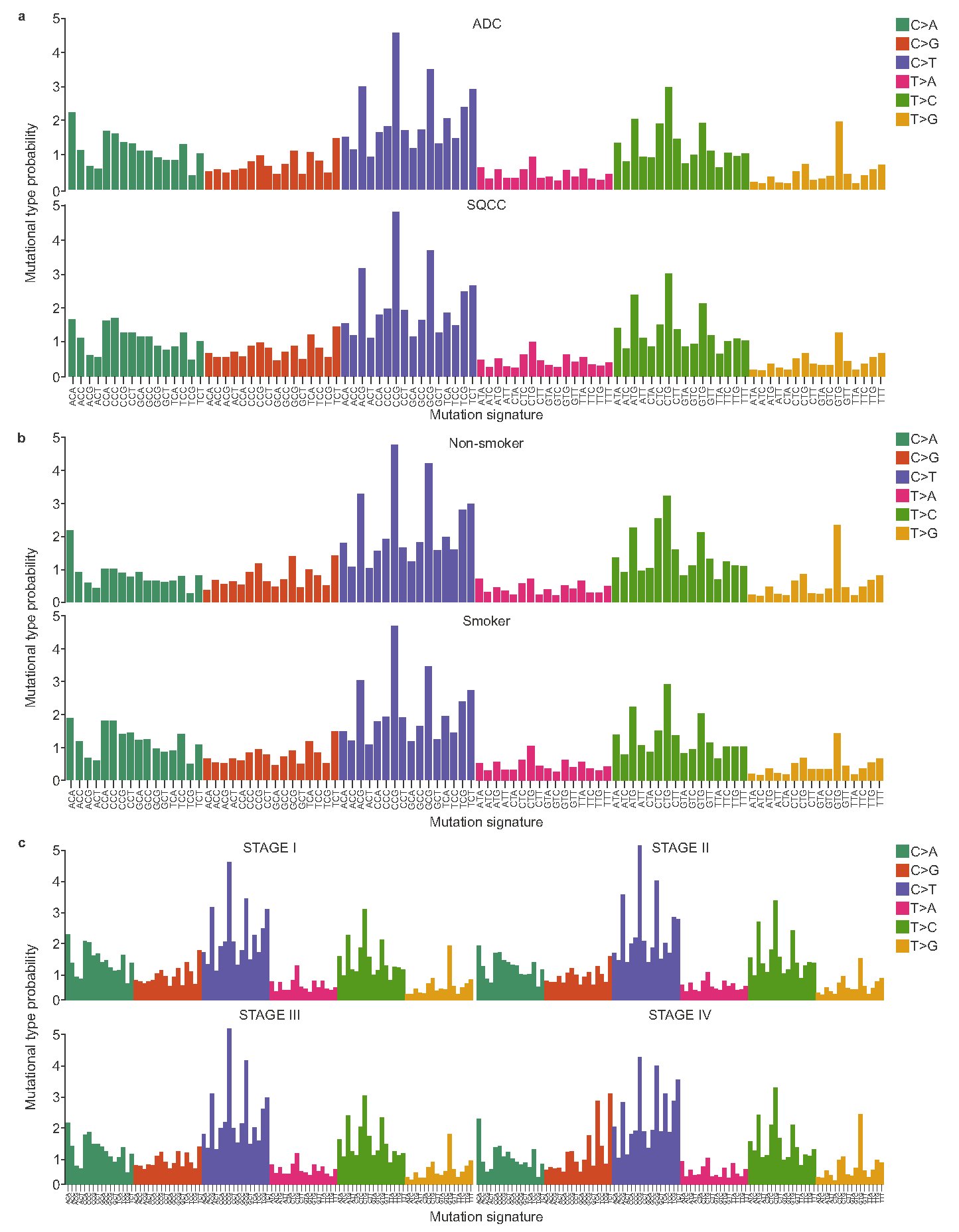
**

**Supplementary Figure 5. Mutational signature profile of the size pyrimidine substitution subtypes. (a) Smoker versus non-smoker. (b) Adenocarcinoma versus squamous cell carcinoma. (c) Stage.**

**
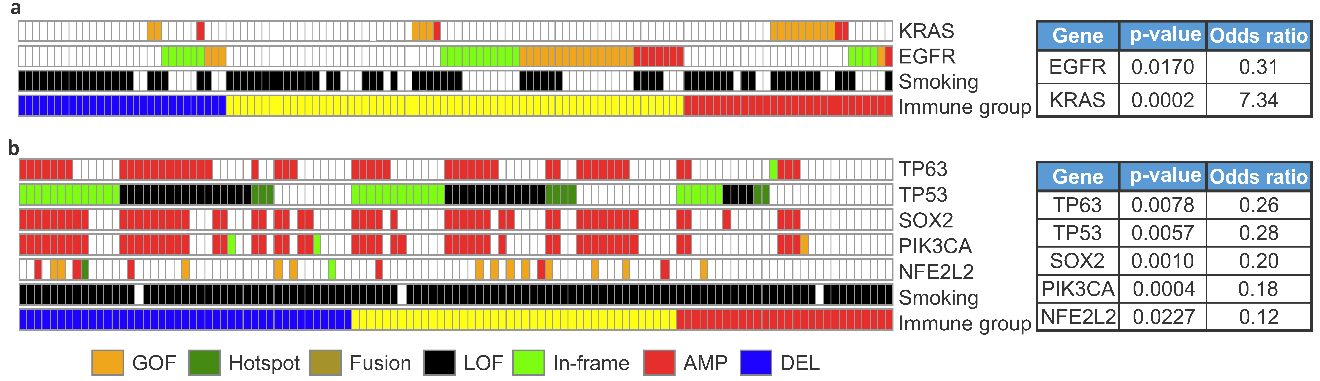
**

**Supplementary Figure 6. Enrichment of driver mutations in immune HIGH sub-population in ADC and SQCC (Immune group: red, yellow and blue for HIGH, MIX and LOW). Different types of genomic alternation are shown in different colors. Each column represents a single patient. When multiple genomic alternations exist in the same sample, AMP/DEL are given high priority to be shown. To conduct fisher exact test, immune status was re-classified into 2 categories: immune HIGH versus the rest. Test p value and odds ratio are shown in the table when comparing mutation versus wild type between smokers and non-smokers. (a) Significant driver mutation in ADC. (b) Significant driver mutation in SQCC.**

**
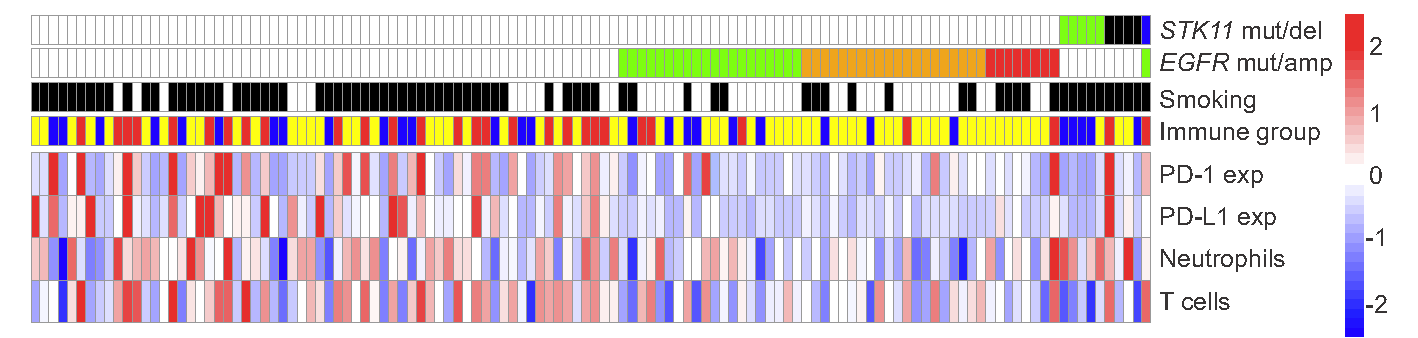
Supplementary Figure 7. In ADC patient samples, STK11 mutation was found to be associated with relatively low T cells signatures (t-test p-value=0.01) and enriched for relatively high neutrophils signature scores (p-value=0.14) comparing with STK11 wild type (n=122).**

**
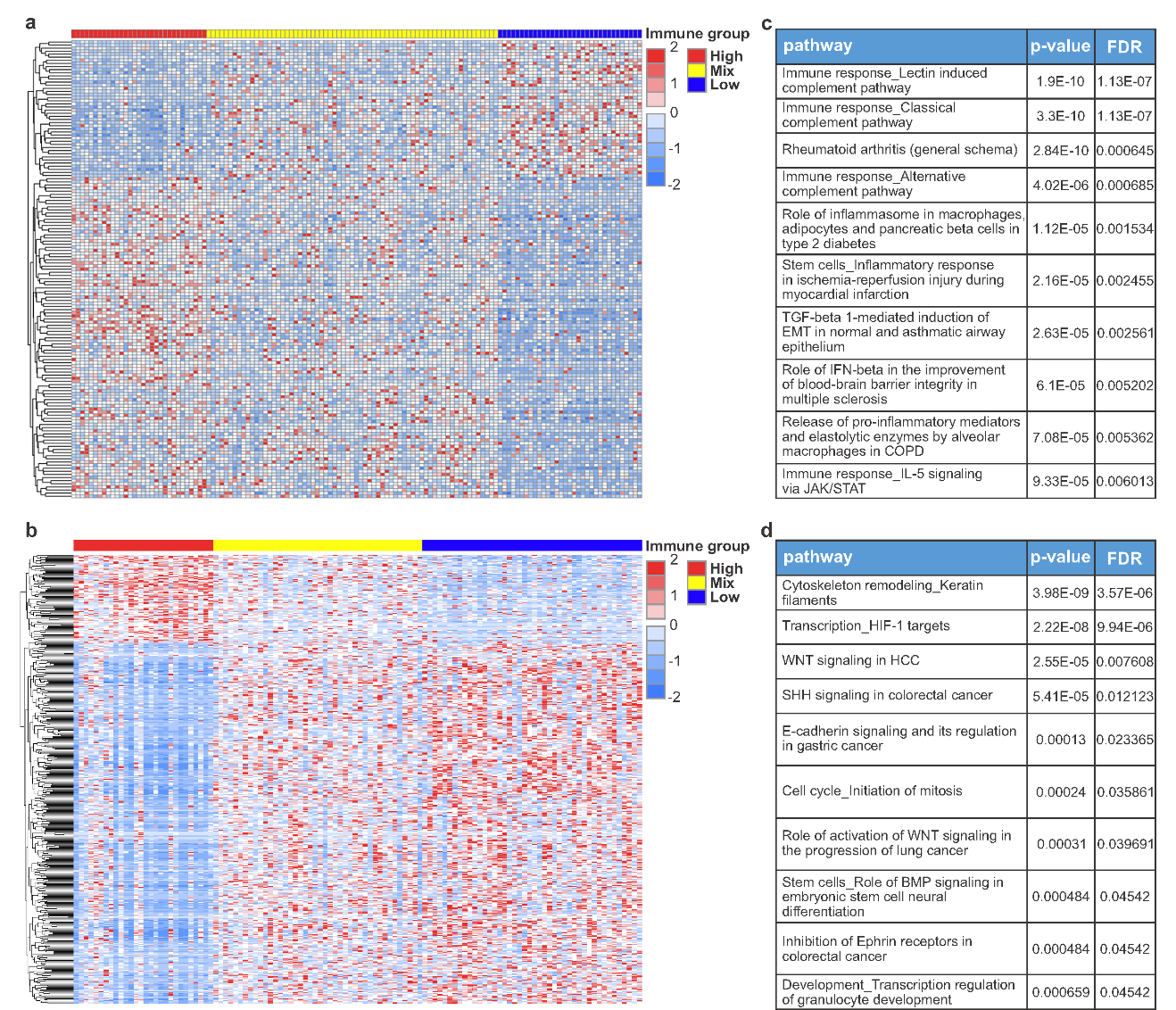
**

**Supplementary Figure 8. Heat map of differentially expressed genes among 3 immune groups. (a) ADC. (b) SQCC. Genes that were highly correlated with immune signatures were removed as detailed in the online method. Pathway enrichment analysis was performed using Metacore to identify differently expressed pathways/gene sets in ADC and SQCC, respectively. (c) Top 10 enriched pathways in ADC. (d) Top 10 enriched pathways in SQCC.**

**
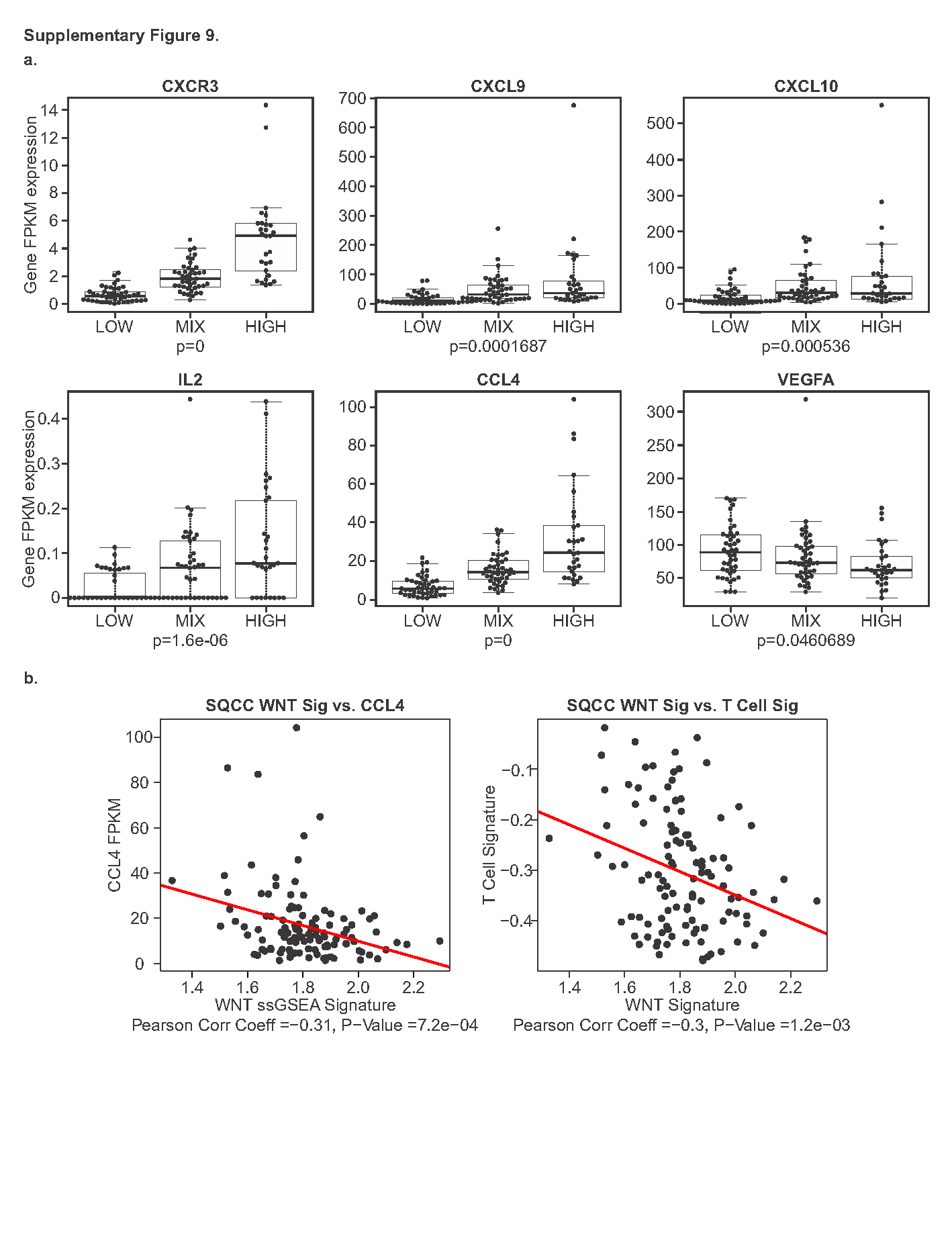
**

**Supplementary Figure 9. (a) Significantly differentially expressed cytokines among immune LOW, MIX and HIGH groups in SQCC. (b) WNT pathway signature calculated using ssGSEA negatively correlated with CCL4 mRNA expression (left) and T cell signatures (right). The top and bottom of the boxes are the lower and upper quartiles. The middle line in the box is median, and the whiskers are lowest and highest point within 1.5 times the interquartile range of the lower and upper quartile.** Source data are provided as a Source Data file**.**

**Supplementary Figure 10. Correlation between PDL1 (CD274) mRNA expression with T cell immune signature In ADC patient samples.** Source data are provided as a Source Data file**.**

**
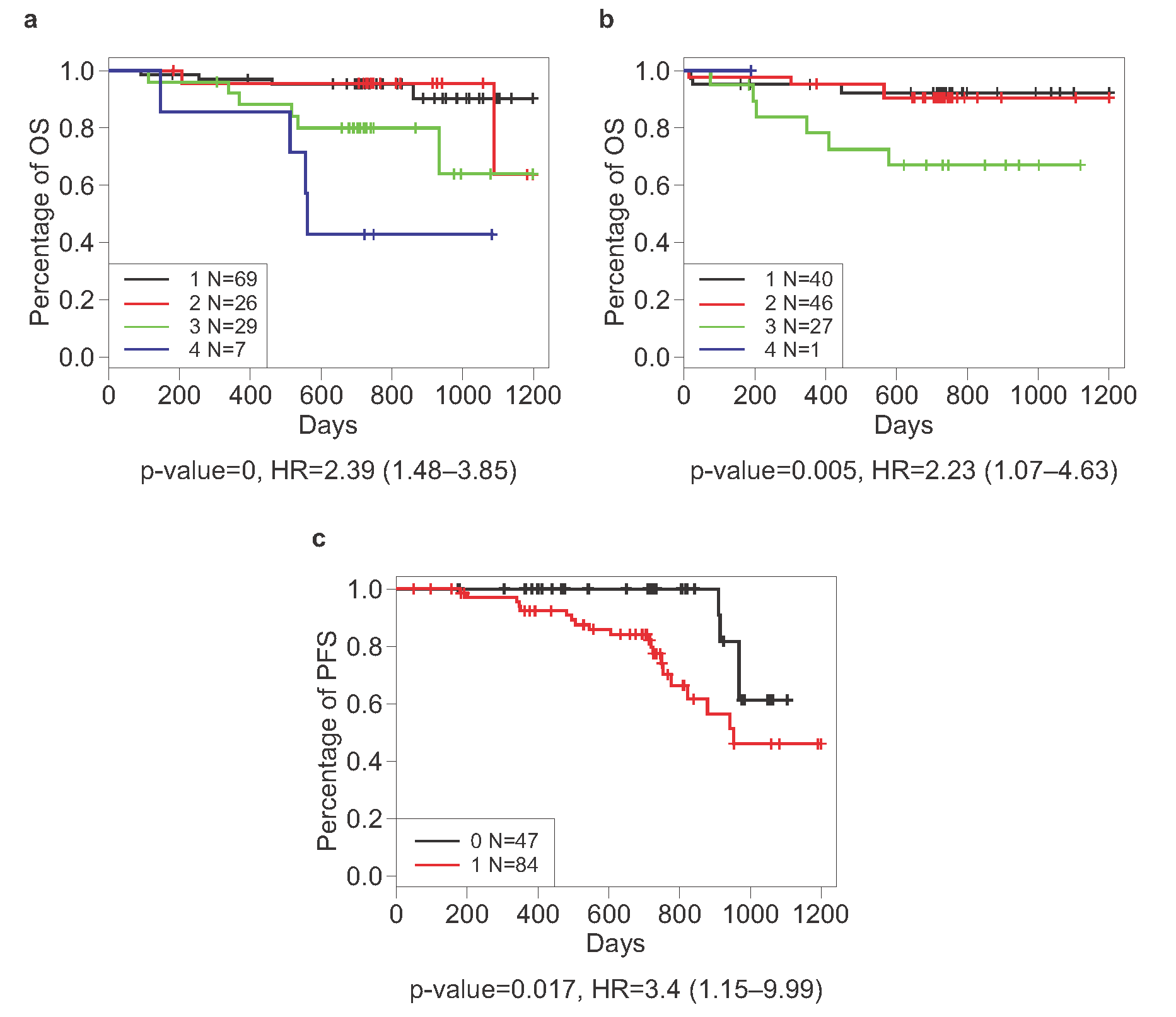
Supplementary Figure 11. Clinical variables that significantly associated with patient survival data. (a) Tumor stage versus OS in ADC. (b) Tumor stage versus OS in SQCC. (c) Smoking status versus PFS in ADC.**
