# Supplementary material for: Comprehensive genomic and immunological characterization of Chinese non-small cell lung cancer patients
Source: Nat Commun. 2019 Apr 16;10:1772. doi: 10.1038/s41467-019-09762-1 (PMC6467893; doi:10.1038/s41467-019-09762-1)
Supplement: Supplementary file 2 — Description of Additional Supplementary Files [file 41467_2019_9762_MOESM2_ESM.pdf]

Description of Additional Supplementary Information

File Name: Supplementary Data 1
Description: CHOICE sample data and patient clinical data

File Name: Supplementary Data 2
Description: CNV data generated from GISTIC2

File Name: Supplementary Data 3
Description: MutSigCV significant gene mutation of ADC tumors

File Name: Supplementary Data 4
Description: MutSigCV significant gene mutation of SQCC tumors

File Name: Supplementary Data 5
Description: Mutation frequency comparison of TCGA and CHOICE ADC tumors

File Name: Supplementary Data 6
Description Mutation frequency comparison of TCGA and CHOICE SQCC tumors

File Name: Supplementary Data 7
Description: Fusion results summary

File Name: Supplementary Data 8
Description: Immune signature score calculated using ssGSEA

File Name: Supplementary Data 9
Description Survival analysis results of immune signatures

File Name: Supplementary Data 10
Description: ADC tumor mutation calls MAF

File Name: Supplementary Data 11
Description: SQCC tumor mutation calls MA
